# Supplementary material for: Crosstalk of Brain and Bone—Clinical Observations and Their Molecular Bases
Source: Int J Mol Sci. 2020 Jul 13;21(14):4946. doi: 10.3390/ijms21144946 (PMC7404044; doi:10.3390/ijms21144946)
Supplement: Supplementary file 1 [file ijms-21-04946-s001.pdf]

Supplementary table 1. Selection of genetic diseases affecting bone and brain

	disorder	disorder	effect on brain and bone	studies/reviews
**brain and bone**	*genetic*	hereditary inclusion body myopathy (IBMPFD)	- associated with paget disease of bone (PDB) and frontotemporal dementia (FTD) is a progressive autosomal dominant that manifests as one or both diseases associated, caused by valosin-containing protein (VCP) mutations<br>- **brain**: 31% of patients develop FTD characterized with antisocial behavior, language dysfunction up to cortical degenerative changes<br>- **bone**: 51% of patients show osteolytic lesions with increased and disorganized bone remodeling consistent with PDB	[38]<br>[39]<br>[40]
		Lenz-Majewski syndrom (LMS)	- heterozygous mutations in the *PTDSS1* gene encoding PSS1, one of the two enzymes involved in the production of phosphatidylserine<br>- **brain**: intellectual disability<br>- **bone**: progressive generalized craniotubular hyperostosis with distinct craniofacial, dental, cutaneous and limb anomalies	[41]
		Nasu-Hakola Disease (NHD or PLOSL)	- also called polycystic lipomembranous osteodysplasia with sclerosing leuko-encephalopathy (PLOSL), caused by *TYROBP* (*DAP12*) and *TREM2* mutations<br>- **brain**: associated with a special form of neurodegeneration that leads to dementia (approx. 4th decade of life) and death (approx. 5th decade of life)<br>- **bone**: characterized by multiple bone cysts leading to Fx	[42]
		tricho-rhino-phalangeal syndrome (TRPS)	- autosomal dominant deletion on chromosome 8, TRPS I (pathogenic variant of *TRPS1*) and TRPS II (deletion of *TRPS1, RAD21* and *EXT1*)<br>- **brain**: intellectual disability is uncommon in TRPS I, but in most cases of TRPS II with typically mild to moderate in severity<br>- **bone**: both types characterized by distinctive facial features, short stature and skeletal anomalies, only TRPS II with multiple osteochondromas	[43]

Supplementary table 2. Selection of genetic polymorphism affecting brain and bone

	gene	effect of genetic polymorphism on brain and/or bone	studies/reviews
**brain**	*CARTPT*	- affects lumbal spine bone mineral density (BMD)	[10]
	VIP	- predicts treatment requirement in early rheumatoid arthritis	[7]
	POMC	- associated with low BMD; haplotypes could be useful markers for predicting antidepressant treatment in major depressive disorder (MDD) patients	[8,9]
	NPY	- polymorphism in neuropeptide Y (NPY) and NPY receptor genes may be useful in identifying women at risk for osteoporosis	[10]
**bone**	*BGLAP*	- may effect the risk of osteoporosis and fracture in a gender dependent manner	[1,2]
	SOST	- associated with BMD, sclerosteosis and van Buchem disease while increased promotor methylation promotes bone formation and may be related to severity of osteogenesis imperfecta	[3-6,44-46]<br>[16,47]
	LRP5	- activating mutations leads to high bone mass while inactivating mutations result in osteoporosis	[48,49]
**brain and bone**	*DKK1*	- multifaceted effects on proliferation and differentiation of various human cells such as osteoblasts and bone marrow cells	[16]
	TNFSF11	- associated with BMD and high risk for Paget's disease	[17-21]
	BDNF	- Val66Met polymorphism presence is a risk factor for psychosis in neurodegenerative diseases such as Alzheimer's and Parkinson's disease	[22-24]
	IGF-1	- significantly associated with BMD and osteoporosis in postmenopausal woman, suggesting rs35767 as predictive factor for osteoporosis risk	[25-29]
	BMP2	- controversy results show different BMD alterations; associated with ossification of the posterior longitudinal ligament	[30-34]
	BMP4	- polymorphism contributes to risk of non-syndromic cleft lip with or without cleft palate and influences early marginal bone loss around implants	[35,36]
	BMP7	- associated with inverse relationships between bone mineralization and vascular calcification	[37]

Supplementary references

1. Ling, Y.; Gao, X.; Lin, H.; Ma, H.; Pan, B.; Gao, J. A common polymorphism rs1800247 in osteocalcin gene was associated with serum osteocalcin levels, bone mineral density, and fracture: the Shanghai Changfeng Study. *Osteoporos. Int.* **2016**, *27*, 769–779, doi:10.1007/s00198-015-3244-5.
2. McGuigan, F.; Kumar, J.; Ivaska, K.K.; Obrant, K.J.; Gerdhem, P.; Åkesson, K. Osteocalcin gene polymorphisms influence concentration of serum osteocalcin and enhance fracture identification. *J. Bone Miner. Res.* **2010**, *25*, 1392–1399, doi:10.1002/jbmr.32.
3. Reppe, S.; Noer, A.; Grimholt, R.M.; Halldórsson, B. V.; Medina-Gomez, C.; Gautvik, V.T.; Olstad, O.K.; Berg, J.P.; Datta, H.; Estrada, K.; et al. Methylation of bone SOST, its mRNA, and serum sclerostin levels correlate strongly with fracture risk in postmenopausal women. *J. Bone Miner. Res.* **2015**, *30*, 249–256, doi:10.1002/jbmr.2342.
4. Delgado-Calle, J.; Sañudo, C.; Bolado, A.; Fernández, A.F.; Arozamena, J.; Pascual-Carra, M.A.; Rodriguez-Rey, J.C.; Fraga, M.F.; Bonewald, L.; Riancho, J.A. DNA methylation contributes to the regulation of sclerostin expression in human osteocytes. *J. Bone Miner. Res.* **2012**, *27*, 926–937, doi:10.1002/jbmr.1491.
5. Lhaneche, L.; Hald, J.D.; Domingues, A.; Hannouche, D.; Delepine, M.; Zelenika, D.; Boland, A.; Ostertag, A.; Cohen-Solal, M.; Langdahl, B.L.; et al. Variations of SOST mRNA expression in human bone are associated with DNA polymorphism and DNA methylation in the SOST gene. *Bone* **2016**, *92*, 107–115, doi:10.1016/j.bone.2016.08.004.
6. He, J.; Zhang, H.; Wang, C.; Zhang, Z.; Yue, H.; Hu, W.; Gu, J.; Fu, W.; Hu, Y.; Li, M.; et al. Associations of serum sclerostin and polymorphisms in the SOST gene with bone mineral density and markers of bone metabolism in postmenopausal chinese women. *J. Clin. Endocrinol. Metab.* **2014**, *99*, 665–673, doi:10.1210/jc.2013-2086.

7. Seoane, I. V.; Martínez, C.; García-Vicuña, R.; Ortiz, A.M.; Juarranz, Y.; Talayero, V.C.; González-Álvaro, I.; Gomariz, R.P.; Lamana, A. Vasoactive intestinal peptide gene polymorphisms, associated with its serum levels, predict treatment requirements in early rheumatoid arthritis. *Sci. Rep.* **2018**, *8*, 1–11, doi:10.1038/s41598-018-20400-6.
8. Chang, H.S.; Won, E.S.; Lee, H.Y.; Ham, B.J.; Kim, Y.G.; Lee, M.S. The association of proopiomelanocortin polymorphisms with the risk of major depressive disorder and the response to antidepressants via interactions with stressful life events. *J. Neural Transm.* **2015**, *122*, 59–68, doi:10.1007/s00702-014-1333-9.
9. Sudo, Y.; Ezura, Y.; Kajita, M.; Yoshida, H.; Suzuki, T.; Hosoi, T.; Inoue, S.; Shiraki, M.; Ito, H.; Emi, M. Association of single nucleotide polymorphisms in the promoter region of the pro-opiomelanocortin gene (POMC) with low bone mineral density in adult women. *J. Hum. Genet.* **2005**, *50*, 235–240, doi:10.1007/s10038-005-0244-x.
10. Chun, E.H.; Kim, H.; Suh, C.S.; Kim, J.H.; Kim, D.Y.; Kim, J.G. Polymorphisms in neuropeptide genes and bone mineral density in Korean postmenopausal women. *Menopause* **2015**, *22*, 1256–1263, doi:10.1097/GME.0000000000000454.
11. Dennison, E.M.; Syddall, H.E.; Rodriguez, S.; Voropanov, A.; Day, I.N.M.; Cooper, C. Polymorphism in the growth hormone gene, weight in infancy, and adult bone mass. *J. Clin. Endocrinol. Metab.* **2004**, *89*, 4898–4903, doi:10.1210/jc.2004-0151.
12. Kenth, G.; Shao, Z.; Cole, D.E.C.; Goodyer, C.G. Relationship of the human growth hormone receptor exon 3 genotype with final adult height and bone mineral density. *J. Clin. Endocrinol. Metab.* **2007**, *92*, 725–728, doi:10.1210/jc.2006-1695.
13. Kripke, D.F.; Nievergelt, C.M.; Tranah, G.J.; Murray, S.S.; McCarthy, M.J.; Rex, K.M.; Parimi, N.; Kelsoe, J.R. Polymorphisms in melatonin synthesis pathways: Possible influences on depression. *J. Circadian Rhythms* **2011**, *9*, 8, doi:10.1186/1740-3391-9-8.
14. Gałecki, P.; Szemraj, J.; Bartosz, G.; Bieńkiewicz, M.; Gałecka, E.; Florkowski, A.; Lewiński, A.; Karbownik-Lewińska, M. Single-nucleotide polymorphisms and mRNA expression for melatonin synthesis rate-limiting enzyme in recurrent depressive disorder. *J. Pineal Res.* **2010**, *48*, 311–317, doi:10.1111/j.1600-079X.2010.00754.x.
15. Etain, B.; Dumaine, A.; Bellivier, F.; Pagan, C.; Francelle, L.; Goubran-botros, H.; Moreno, S.; Deshommes, J.; Moustafa, K.; Le dudal, K.; et al. Genetic and functional abnormalities of the melatonin biosynthesis pathway in patients with bipolar disorder. *Hum. Mol. Genet.* **2012**, *21*, 4030–4037, doi:10.1093/hmg/dds227
16. Sharma, G.; Sharma, A.R.; Seo, E.M.; Nam, J.S. Genetic polymorphism in extracellular regulators of Wnt signaling pathway. *Biomed Res. Int.* **2015**, *2015*, doi:10.1155/2015/847529.
17. Mencej, S.; Preželj, J.; Kocijančič, A.; Ostanek, B.; Marc, J. Association of TNFSF11 gene promoter polymorphisms with bone mineral density in postmenopausal women. *Maturitas* **2006**, *55*, 219–226, doi:10.1016/j.maturitas.2006.03.004.
18. Tu, P.; Duan, P.; Zhang, R.S.; Xu, D.B.; Wang, Y.; Wu, H.P.; Liu, Y.H.; Si, L. Polymorphisms in genes in the RANKL/RANK/OPG pathway are associated with bone mineral density at different skeletal sites in post-menopausal women. *Osteoporos. Int.* **2014**, *26*, 179–185, doi:10.1007/s00198-014-2854-7.
19. Chung, P.Y.J.; Beyens, G.; Boonen, S.; Papapoulos, S.; Geusens, P.; Karperien, M.; Vanhoenacker, F.; Verbruggen, L.; Fransen, E.; Van Offel, J.; et al. The majority of the genetic risk for Paget's disease of bone is explained by genetic variants close to the CSF1, OPTN, TM7SF4, and TNFRSF11A genes. *Hum. Genet.* **2010**, *128*, 615–626, doi:10.1007/s00439-010-0888-2.
20. Zupan, J.; Mencej-Bedrač, S.; Jurković-Mlakar, S.; Preželj, J.; Marc, J. Gene-gene interactions in RANK/RANKL/OPG system influence bone mineral density in postmenopausal women. *J. Steroid Biochem. Mol. Biol.* **2010**, *118*, 102–106, doi:10.1016/j.jsbmb.2009.10.013.
21. Hughes, A.E.; Ralston, S.H.; Marken, J.; Bell, C.; MacPherson, H.; Wallace, R.G.H.; Van Hul, W.; Whyte, M.P.; Nakatsuka, K.; Hovy, L.; et al. Mutations in TNFRSF11A, affecting the signal peptide of RANK, cause familial expansile osteolysis. *Nat. Genet.* **2000**, *24*, 45–48, doi:10.1038/71667.
22. Shen, T.; You, Y.; Joseph, C.; Mirzaei, M.; Klistorner, A.; Graham, S.L.; Gupta, V. BDNF polymorphism: A review of its diagnostic and clinical relevance in neurodegenerative disorders. *Aging Dis.* **2018**, *9*, 523–536, doi:10.14336/AD.2017.0717.
23. Pivac, N.; Nikolac, M.; Nedic, G.; Mustapic, M.; Borovecki, F.; Hajnsek, S.; Presecki, P.; Pavlovic, M.; Mimica, N.; Muck Seler, D. Brain derived neurotrophic factor Val66Met polymorphism and psychotic symptoms in Alzheimer's disease. *Prog. Neuro-Psychopharmacology Biol. Psychiatry* **2011**, *35*, 356–362, doi:10.1016/j.pnpbp.2010.10.020.
24. Park, C.H.; Kim, J.; Namgung, E.; Lee, D.W.; Kim, G.H.; Kim, M.; Kim, N.; Kim, T.D.; Kim, S.; Lyoo, I.K.; et al. The BDNF val66met polymorphism affects the vulnerability of the brain structural network. *Front. Hum. Neurosci.* **2017**, *11*, 1–10, doi:10.3389/fnhum.2017.00400.
25. Yun-Kai, L.; Hui, W.; Xin-Wei, Z.; Liang, G.; Jin-Liang, Z. The polymorphism of insulin-like growth factor-l (IGF-I) is related to osteoporosis and bone mineral density in postmenopausal population. *Pakistan J. Med. Sci.* **2014**, *30*, 131–135, doi:10.12669/pjms.301.4264.
26. Li, F.; Xing, W.H.; Yang, X.J.; Jiang, H.Y.; Xia, H. Influence of polymorphisms in insulin-like growth factor-1 on the risk of osteoporosis in a Chinese postmenopausal female population. *Int. J. Clin. Exp. Pathol.* **2015**, *8*, 5727–5732.
27. Zhang, W.; Zhang, L.C.; Chen, H.; Tang, P.F.; Zhang, L.H. Association between polymorphisms in insulin-like growth factor-1 and risk of osteoporosis. *Genet. Mol. Res.* **2015**, *14*, 7655–7660, doi:10.4238/2015.July.13.10.
28. Chen, Y.C.; Zhang, L.; Li, E.N.; DIng, L.X.; Zhang, G.A.; Hou, Y.; Yuan, W. Association of the insulin-like growth factor-1 single nucleotide polymorphisms rs35767, rs2288377, and rs5742612 with osteoporosis risk. *Med. (United States)* **2017**, *96*, 1–6, doi:10.1097/MD.0000000000009231.

29. Gao, S.T.; Lv, Z.T.; Zhou, C.K.; Mao, C.; Sheng, W. Bin Association between IGF-1 polymorphisms and risk of osteoporosis in Chinese population: A meta-analysis. *BMC Musculoskelet. Disord.* **2018**, *19*, 1–8, doi:10.1186/s12891-018-2066-y.
30. Wang, H.; Liu, D.; Yang, Z.; Tian, B.; Li, J.; Meng, X.; Wang, Z.; Yang, H.; Lin, X. Association of bone morphogenetic protein-2 gene polymorphisms with susceptibility to ossification of the posterior longitudinal ligament of the spine and its severity in Chinese patients. *Eur. Spine J.* **2008**, *17*, 956–964, doi:10.1007/s00586-008-0651-8.
31. Fritz, D.T.; Jiang, S.; Xu, J.; Rogers, M.B. A polymorphism in a conserved posttranscriptional regulatory motif alters bone morphogenetic protein 2 (BMP2) RNA:Protein interactions. *Mol. Endocrinol.* **2006**, *20*, 1574–1586, doi:10.1210/me.2005-0469.
32. Ichikawa, S.; Johnson, M.L.; Koller, D.L.; Lai, D.; Xuei, X.; Edenberg, H.J.; Hui, S.L.; Foroud, T.M.; Peacock, M.; Econs, M.J. Polymorphisms in the bone morphogenetic protein 2 (BMP2) gene do not affect bone mineral density in white men or women. *Osteoporos. Int.* **2006**, *17*, 587–592, doi:10.1007/s00198-005-0018-5.
33. Ozkan, Z.S.; Deveci, D.; Onalan Etem, E.; Yüce, H. Lack of effect of bone morphogenetic protein 2 and 4 gene polymorphisms on bone density in postmenopausal Turkish women. *Genet. Mol. Res.* **2010**, *9*, 2311–2316, doi:10.4238/vol9-4gmr922.
34. Choi, J.Y.; Shin, C.S.; Hong, Y.C.; Kang, D. Single-nucleotide polymorphisms and haplotypes of bone morphogenetic protein genes and peripheral bone mineral density in young Korean men and women. *Calcif. Tissue Int.* **2006**, *78*, 203–211, doi:10.1007/s00223-005-0139-z.
35. Lin, J.Y.; Chen, Y.J.; Huang, Y.L.; Tang, G.P.; Zhang, L.; Deng, B.; Li, M.; Ma, H.; Luan, R.S. Association of bone morphogenetic protein 4 gene polymorphisms with nonsyndromic cleft lip with or without cleft palate in Chinese children. *DNA Cell Biol.* **2008**, *27*, 601–605, doi:10.1089/dna.2008.0777.
36. Shimpuku, H.; Nosaka, Y.; Kawamura, T.; Tach, Y.; Shinohara, M.; Ohura, K. Bone morphogenetic protein-4 gene polymorphism and early marginal bone loss around endosseous implants. *Int. J. Oral Maxillofac. Implant.* **2003**, *18*, 500–504.
37. Freedman, B.I.; Bowden, D.W.; Ziegler, J.T.; Langefeld, C.D.; Lehtinen, A.B.; Rudock, M.E.; Lenchik, L.; Hruska, K.A.; Register, T.C.; Carr, J.J. Bone morphogenetic protein 7 (BMP7) gene polymorphisms are associated with inverse relationships between vascular calcification and BMD: The diabetes heart study. *J. Bone Miner. Res.* **2009**, *24*, 1719–1727, doi:10.1359/jbmr.090501.
38. Watts, G.D.J.; Wymer, J.; Kovach, M.J.; Mehta, S.G.; Mumm, S.; Darvish, D.; Pestronk, A.; Whyte, M.P.; Kimonis, V.E. Inclusion body myopathy associated with Paget disease of bone and frontotemporal dementia is caused by mutant valosin-containing protein. *Nat. Genet.* **2004**, *36*, 377–381, doi:10.1038/ng1332.
39. Kimonis, V.E.; Watts, G.D.J. Autosomal dominant inclusion body myopathy, Paget disease of bone, and frontotemporal dementia. *Alzheimer Dis. Assoc. Disord.* **2005**, *19*, 44–47, doi:10.1097/01.wad.0000183081.76820.5a.
40. Watts, G.D.G.; Thomasova, D.; Ramdeen, S.K.; Fulchiero, E.C.; Mehta, S.G.; Drachman, D.A.; Weihl, C.C.; Jamrozik, Z.; Kwiecinski, H.; Kaminska, A.; et al. Novel VCP mutations in inclusion body myopathy associated with Paget disease of bone and frontotemporal dementia. *Clin. Genet.* **2007**, *72*, 420–426, doi:10.1111/j.1399-0004.2007.00887.x.
41. Sousa, S.B.; Jenkins, D.; Chanudet, E.; Tasseva, G.; Ishida, M.; Anderson, G.; Docker, J.; Ryten, M.; Sa, J.; Saraiva, J.M.; et al. Gain-of-function mutations in the phosphatidylserine synthase 1 (PTDSS1) gene cause Lenz-Majewski syndrome. *Nat. Genet.* **2014**, *46*, 70–76, doi:10.1038/ng.2829.
42. Paloneva, J.; Autti, T.; Raininko, R.; Partanen, J.; Salonen, O.; Puranen, M.; Hakola, P.; Haltia, M. CNS manifestations of Nasu-Hakola disease: A frontal dementia with bone cysts. *Neurology* **2001**, *56*, 1552–1558, doi:10.1212/wnl.56.11.1552.
43. Maas, S.; Shaw, A.; Bikker, H. Trichorhinophalangeal Syndrome Summary GeneReview Scope. *GeneReviews* **2019**, 1–20.
44. Martínez-Gil, N.; Roca-Ayats, N.; Monistrol-Mula, A.; García-Giralt, N.; Díez-Pérez, A.; Nogués, X.; Mellibovsky, L.; Grinberg, D.; Balcells, S. Common and rare variants of WNT16, DKK1 and SOST and their relationship with bone mineral density. *Sci. Rep.* **2018**, *8*, 1–10, doi:10.1038/s41598-018-29242-8.
45. Balemans, W.; Van Hul, W. Identification of the disease-causing gene in sclerosteosis - Discovery of a novel bone anabolic target? *J. Musculoskelet. Neuronal Interact.* **2004**, *4*, 139–142.
46. Balemans, W.; Patel, N.; Ebeling, M.; Van Hul, E.; Wuyts, W.; Lacza, C.; Dioszegi, M.; Dikkers, F.G.; Hildering, P.; Willems, P.J.; et al. Identification of a 52 kb deletion downstream of the SOST gene in patients with van Buchem disease. *J. Med. Genet.* **2002**, *39*, 91–97, doi:10.1136/jmg.39.2.91.
47. De Ridder, R.; Boudin, E.; Mortier, G.; Van Hul, W. Human Genetics of Sclerosing Bone Disorders. *Curr. Osteoporos. Rep.* **2018**, *16*, 256–268, doi:10.1007/s11914-018-0439-7.
48. Little, R.D.; Recker, R.R.; Johnson, M.L.; Hofbauer, L.C.; Maisch, B.; Schaefer, J.R.; Boyden, L.; Insogna, K.; Lifton, R.P. High bone density due to a mutation in LDL-receptor-related protein 5 [1] (multiple letters). *N. Engl. J. Med.* **2002**, *347*, 943–944, doi:10.1056/NEJM200209193471216.
49. Hartikka, H.; Mäkitie, O.; Männikkö, M.; Doria, A.S.; Daneman, A.; Cole, W.G.; Ala-Kokko, L.; Sochett, E.B. Heterozygous mutations in the LDL receptor-related protein 5 (LRP5) gene are associated with primary osteoporosis in children. *J. Bone Miner. Res.* **2005**, *20*, 783–789, doi:10.1359/JBMR.050101.
